# The use of machine learning interatomic potentials for the verification of experimental molecular crystal structures

**DOI:** 10.1107/S2052252526006949

**Published:** 2026-07-27

**Authors:** M. Hušák, F. Fňukal, J. Čejka

**Affiliations:** a Department of Solid State Chemistry University of Chemistry and Technology Technická 5, Praha 6 Prague166 28 Czechia; Sun Yat-Sen University, China

**Keywords:** MLIPs, structure validation, databases, DFT, molecular crystals, machine learning

## Abstract

A correctly solved crystal structure should agree with the experimental data, and its geometry should correspond to a local minimum on the potential energy surface (PES). The idea of verifying crystal structure solutions by comparing them with their geometry-optimized versions was introduced 15 years ago. Recent developments in machine learning interatomic potentials (MLIPs) have made it possible to replace computationally expensive density functional theory (DFT) calculations with AI/neural-network-based alternatives. MLIPs can reach DFT-comparable precision with a substantial gain in speed. We selected one promising MLIP, Universal Models for Atoms, trained on the Open Molecular Crystals 2025 dataset, and processed a prefiltered subset of 216 919 structures from the Cambridge Structural Database. Due to the limitations of the MLIP available when this study commenced, ionic compounds, salts and metal-containing structures were excluded. The current methodology cannot process disordered structures, and available computational resources limit the maximum unit-cell volume that can be treated to 4000 Å^3^. All structures in the dataset were geometry optimized using the MLIP, and similarity descriptors were calculated to quantify the differences between the original and optimized structures. Automatic analysis was followed by the manual identification of issues indicated by the descriptors’ values. We detected anomalies in experimental structures that had already passed all prior validation, as well as limitations in the reliability of the MLIP PES calculations. For 1867 crystal structures, bond-pattern change was observed, while 3331 structures showed a root-mean-square Cartesian displacement greater than 0.25 Å. Future improvements to the methodology and extension to systems not covered by this study are discussed.

## Abstract 2

Machine learning interatomic potentials (MLIPs) offer a significantly faster alternative to density functional theory for theoretical crystal structure calculations with comparable accuracy. Geometry-optimized structures generated using the Universal Models for Atoms MLIP, trained on the Open Molecular Crystals 2025 dataset, were used to verify 216 919 selected structures from the Cambridge Structural Database with the aim of detecting issues in both the interpretation of the original X-ray experiments and the performance of the used MLIP.

## 1. Introduction

Crystal structure validation is typically performed by evaluating agreement with the original experimental data. Such an evaluation is accomplished using tools such as the IUCr checkCIF service, which is based on the *PLATON* code (Spek, 2020 ▸). *PLATON* also provides a limited molecular-geometry check. A more detailed molecular-geometry check can be performed using *CSD Mogul* (Groom *et al.*, 2016 ▸). However, neither approach can verify the ‘chemical sense’ of the structure or the molecular arrangement, as demonstrated by Raymond & Girolami (2023 ▸).

An alternative method was introduced by van de Streek & Neumann (2010 ▸, 2014 ▸) 15 years ago. This method is based on comparing the experimental structure with its geometry-optimized version obtained through lattice-energy minimization. The approach was tested on single-crystal data (van de Streek & Neumann, 2010 ▸) and on powder data (van de Streek & Neumann, 2014 ▸). The energy was calculated using the Perdew–Burke–Ernzerhof (PBE) density functional with dispersion correction. The number of structures processed in this way was limited by computational cost. Density functional theory (DFT)-based structure-geometry optimization remains resource-intensive, typically requiring several hours or days, as demonstrated during the seventh crystal structure prediction blind test (Hunnisett *et al.*, 2024 ▸). This speed is sufficient for checking individual structures but not for large-scale data checking or online structure verification services. The key crystallographic databases – namely, the Cambridge Structural Database (CSD; Groom *et al.*, 2016 ▸), the Inorganic Crystal Structure Database (ICSD; Levin, 2018 ▸) and the Crystallography Open Database (COD; Gražulis *et al.*, 2012 ▸) – together contain more than 2 million structures. Checking this number of structures by DFT would require several years of work on modern supercomputers.

There are three main alternatives to DFT for generating the potential energy surface (PES) required for geometry optimization. An overview and benchmark of these methods are provided in the article describing the Open Force Field (OFF) parameterization of the multiplicative atom cluster expansion (MACE) engine (Kovács *et al.*, 2025 ▸).

The first alternative consists of empirical molecular mechanics (MM) force fields, such as the general AMBER force field (Wang *et al.*, 2004 ▸) or Sage (Boothroyd *et al.*, 2023 ▸). Empirical MM force fields are applicable to a wide range of compounds and are computationally fast, but the accuracy and precision of the results are not sufficient for crystal structure verification. To improve performance, the force field must be tailored to the given system; however, this typically requires DFT calculations to obtain calibration data, and, therefore, does not reduce the overall computational demands.

Semi-empirical methods based on density functional tight binding (DFTB), such as GFN2-xTB (Bannwarth *et al.*, 2019 ▸), provide a second alternative. These methods are more precise than empirical MM force fields and are parameterized for most elements, but they still do not reach the accuracy and reliability of DFT-based methods.

The third alternative consists of machine learning inter­atomic potential (MLIP)-based force fields. MLIPs are primarily based on kernels or machine-learning neural networks. A review of existing technologies is provided by Unke *et al.* (2021 ▸). This field is developing rapidly, with new engines and models being published frequently. The principle of MLIPs is to create a training set using a high-level computational method, typically DFT, and then reproduce the parameters of that training set. In the training dataset, the structures are labelled with energies, and each atom is labelled with its associated forces. The MLIP model is then optimized to derive the parameters of unknown systems based on the processing of the training set. The main advantage of this approach is that it can achieve almost the same precision and accuracy as the original high-level computational method at a fraction of the computational cost. In addition, MLIP scales more favourably with the number of atoms *n*, namely *O*(*n*), compared with *O*(*n*^3^) for DFT. However, all problems associated with the original high-level computational method used for model generation are transferred to the MLIP. As shown by Kovács *et al.* (2025 ▸), neither empirical MM force fields nor DFTB methods can compete with MLIPs in terms of precision and accuracy.

To verify the conclusion of Kovács *et al.* (2025 ▸) that MLIPs offer the most suitable compromise between accuracy and computational cost, we performed a benchmark comparing MM, DFTB, MLIP and full DFT calculations. The COMPASS III force field (BIOVIA, 2022 ▸), DFTB+ with the 3ob parameter set (BIOVIA, 2022 ▸) and r^2^SCAN + MBD (Furness *et al.*, 2020 ▸) were used as representatives of MM, DFTB and DFT, respectively. The used dataset selection is described in Section 2.2. As shown in Table S1 of the supporting information, the average root-mean-square Cartesian displacement (RMSCD) (van de Streek & Neumann, 2010 ▸) for both MM and DFTB exceeds 0.25 Å, suggesting that the errors introduced by these methods are above the commonly accepted threshold for detecting problematic crystal structures.

The use of MLIPs has some limitations. They are typically trained on selected sets of compounds and phase types. They give good results for compounds similar to those in the training set, but extrapolation is challenging. Most MLIPs are not trained on periodic structures, which is one reason why they do not correctly describe long-range electrostatic interactions. A second reason for problems with long-range interactions is the distance cut-off used in message-passing neural network (MPNN) technology for force calculations. Because atoms communicate only with other atoms up to a given cut-off distance, the model cannot describe long-range interactions. A further limitation of the method is the requirement that all atoms are included in the crystal structure. Crystal structures with missing hydrogen atoms or solvent molecules cannot be evaluated or are evaluated incorrectly. The evaluation of disordered structures is theoretically possible by generating all possible unit-cell configurations, but this is not yet supported by our processing software.

Our computational resources, as well as the capabilities of existing MLIPs, are limited. Therefore, we focused our study on a selected subset of crystal structures. Because we chose pharmaceutically relevant compounds, we restricted our research to MLIPs and engines suitable for this purpose. The aim was to achieve the best precision of the PES generated by MLIPs, even at the cost of restricted support for a limited number of elements (H, C, N, O, F, P, S, Cl, Br and I) and for closed-shell systems. Consequently, the suitable databases for performing these tests were primarily pure organic subsets of the CSD or COD. Most existing MLIPs do not describe long-range electrostatic interactions well, which is why we also rejected salts and structures with charged atoms. As described in Section 2.2, we tested three engines, MACE (Kovács *et al.*, 2025 ▸), MACELES (Kim *et al.*, 2025 ▸) and Universal Models for Atoms (UMA) (Wood *et al.*, 2026 ▸), using different models and model sizes [small (S), medium (M) and large (L)] as candidates for the main data processing. Because some models were not available in multiple variants or did not fit within the available hardware constraints, a total of eight PES-generating engine setups were benchmarked. MACE, MACELES and UMA are based on MPNN technology and can run very rapidly on current AI accelerators, primarily owing to NVIDIA CUDA technology and the PyTorch library. As described in Section 2.2, the UMA engine, the S-size model and the Open Molecular Crystals 2025 (OMC25) training dataset (Gharakhanyan *et al.*, 2026 ▸) were chosen for the final large-scale computational test.

## 2. Methodology

### 2.1. Selection of CIF for analysis

We used the CSD database 6.00 as the crystallographic information file (CIF) source for the calculations. At the current stage of MLIP technology, it is impossible to apply the procedure to entire databases, which is why we selected a subset of structures that is well covered by the features of the MLIP used. The first round of structure filtering was performed using *ConQuest* 2025.1.0 (Bruno *et al.*, 2002 ▸). The first restriction concerned the elements present. Structures containing only H, C, N, O, F, P, S, Cl, Br and I were allowed. Because MLIPs available at the study onset did not work correctly with long-range interactions, all structures with ionized fragments or charged atoms were rejected. The other selection criteria were as follows: no disorder, no errors, not polymeric, 3D coordinates present, single-crystal data, and publication after 2004. The latter criterion was intended primarily to include only structures measured using modern diffractometers.

The second round of filtering was performed using our structure verification code, *checkCIF-DFT* (Fňukal, 2024 ▸), because it could not be performed directly in *ConQuest*. Structures with cell volumes greater than 4000 Å^3^ were rejected because of our limited computational resources, as processing these structures exceeded the GPU memory of the AI accelerator used. We also rejected structures containing solvent-accessible volumes greater than 40 Å^3^, because these typically contain undescribed solvents. Another check was performed for potentially incorrectly determined space groups or unnecessarily used supercells. This verification was performed using the *checkCIF-DFT* code interface to the SPGLIB library (Togo *et al.*, 2024 ▸). We are aware that the space-group check and supercell check can generate false alerts, and that confirmation would require analysis of the original data; however, this is outside the scope of this study. The remaining structures were checked for elemental valence. Structures with chemically unreasonable valences were rejected, typically because of missing hydrogen atoms. The final round of rejection was based on the detection of ‘?’ in atom labels. Because CSD does not mark structures with disordered H atoms as disordered, all structures with any disordered atoms (indicated by labels containing ‘?’) had to be rejected. After filtering, 216 919 structures suitable for checking remained. Table 1 ▸ shows the number of structures retained after each filtering step.

We also considered analysing the content of the COD (Gražulis *et al.*, 2012 ▸) in addition to the CSD. The COD (as of 2 July 2025) contains 122 487 structures that satisfy the inclusion criteria (composition, absence of disorder, presence of 3D coordinates and publication year). However, the COD does not include descriptors for identifying ionization states. Therefore, analysis of COD data will become meaningful once MLIPs capable of correctly handling ions are available or once reliable automatic identification of ionized structures in the COD becomes possible.

### 2.2. Benchmarking available MLIP engines

The selection of the most suitable MLIP for large-scale testing was based on both a literature survey and our own benchmarking. A promising candidate previously tested for crystal structure prediction (Nickerson & Johnson, 2025 ▸) was the MACE engine (Kovács *et al.*, 2025 ▸) with the OFF24(M) model. We also tested the MACE engine modified with the Latent Ewald Summation (LES) extension to improve the description of electrostatic interactions; this variant is referred to as MACELES (Kim *et al.*, 2025 ▸). Both the MACE and MACELES models are primarily trained on the SPICE dataset (Eastman *et al.*, 2024 ▸). As a state-of-the-art alternative, we also evaluated the UMA engine developed by the Meta AI research group using two model sizes (S and M) trained on the OMC25 dataset (Gharakhanyan *et al.*, 2026 ▸).

The criteria for engine selection were coverage of pharmaceutically relevant elements (see Section 2.1), computational speed, and the RMSCD (van de Streek & Neumann, 2010 ▸) between test structures and structures optimized by the engine.

Several existing benchmark datasets were considered for evaluating the MLIP. The X23b dataset (Dolgonos *et al.*, 2019 ▸) was not selected because it consists primarily of relatively simple molecular crystals and was considered insufficiently representative of the chemical diversity targeted in this study. We also evaluated the more recent CPOSS209 benchmark set (Price *et al.*, 2025 ▸), which contains both experimentally observed polymorphs and hypothetical structures generated by crystal structure prediction. While CPOSS209 provides a valuable and challenging benchmark for crystal-energy ranking methods, a substantial fraction of the dataset comprises hypothetical structures whose experimental relevance remains uncertain. In addition, the reference structures were optimized using periodic DFT at the PBE + TS level of theory. Given the known limitations of the PBE functional, particularly its self-interaction errors, we decided not to use CPOSS209 as the primary benchmark dataset in the present work.

We ultimately constructed a benchmark set primarily focused on structures with the most reliable structure determinations. In a parallel study on DFT-based structure verification, we previously generated a dataset of 1000 semi-randomly sampled entries from the CSD, for which r^2^SCAN + MBD geometry optimizations were performed using *CASTEP* 23.1 (Clark *et al.*, 2005 ▸). From this dataset, a subset of 20 structures was selected using the following criteria: *R* factor < 0.05, full deposition of *F*_obs_ data in the CSD, RMSCD between the experimental and DFT-optimized structures below 0.2 Å, and satisfaction of all other criteria described in Section 2.1. We acknowledge that this selection strategy, which prioritizes structural correctness over diversity, does not provide a balanced representation of molecular size, space groups, functional groups or hydrogen-bonding motifs. A future larger benchmark set will need in addition to the focus on accuracy and correctness to be focused on structure diversity.

As the benchmark reference and starting point for the calculations, we used the DFT-optimized structures rather than the original experimental ones. This step was necessary to minimize the influence of experiment-dependent atomic position errors on the RMSCD calculation. The results are provided in Table 2 ▸.

Because the calculation speed depended primarily on the AI accelerator’s GPU memory limitations (our hardware limit was 24 GB), we were restricted to small or medium-sized models. Larger models typically yield higher accuracy (see average MACE OFF23 M, L, S RMSCD in Table 2 ▸), but they limit the size of structures that can be processed within reasonable time. Large models combined with large structures may not fit into 24 GB of GPU memory, resulting in shared memory usage between CPU and GPU. This reduces performance by approximately two orders of magnitude. As a result, large models applied to large unit cells require dedicated AI accelerators beyond the scope of our available hardware resources.

The final choice of model used was the UMA (small-S) model trained on the OMC25 dataset (Gharakhanyan *et al.*, 2026 ▸). This model provided the best average RMSCD agreement with the benchmark structures. The OMC25 dataset consists of over 27 million molecular crystal structures containing 12 elements and up to 300 atoms per unit cell. These structures were artificially generated from the OE62 dataset (Stuke *et al.*, 2020 ▸). The statistics of structural motifs and element occurrence frequencies for neither OMC25 nor OE62 have been published; however, because OMC25 is derived from OE62, similar distributions are expected. Based on CSD refcodes provided as part of the OE62 supplementary data, we compiled a basic overview of elemental frequencies and selected rare bond-type occurrences within this dataset (Table S2). The OMC25 dataset uses plane-wave DFT with the PBE-D3 functional to compute energies and forces and is thus expected to reproduce results similar to those obtained from full DFT calculations using this functional with dispersion correction. As described in the original article (Gharakhanyan *et al.*, 2026 ▸), atomic positions and unit-cell parameters were relaxed until the maximum per-atom residual force fell below 0.001 eV Å^−1^ or until 1500 relaxation steps were reached. The total-energy convergence criterion was set to 0.001 meV and the plane-wave energy cut-off was 520 eV. The *k*-point grids were generated automatically using the Pymatgen library. In addition to the final relaxed structures, the dataset contains structures sampled along the relaxation trajectories to represent non-equilibrium states.

### 2.3. Performing large-scale calculations

All calculations were managed using our in-house software, *checkCIF-DFT* (Fňukal, 2024 ▸). The code was originally developed for crystal structure validation using the DFT and MM methods, and its adaptation for MLIP-based calculations was straightforward. The *checkCIF-DFT* code reads the input CIF, generates the complete unit-cell content using symmetry operations, transforms atomic coordinates to the [0, 1) fractional coordinate interval, and exports the structure as a CIF in the *P*1 space group. The individual translations required to shift atoms into the exported unit cell are recorded so that the optimized structures can later be transformed back to the form contained in the original CIF.

The exported CIF is then processed using the Atomic Simulation Environment (ASE) (Hjorth Larsen *et al.*, 2017 ▸). The ASE can use the *MACECalculator* (interface to MACE and MACELES) or the *FAIRChemCalculator* (interface to UMA) plug-in as internal calculators for force evaluation. It supports both CPU-based execution and CUDA-accelerated GPU execution via the PyTorch interface. All calculations were performed using single-precision (float32) arithmetic and GPU execution. Both the unit-cell parameters and all atomic coordinates were refined. The number of optimization steps was limited to 1000. Optimization was considered complete when the forces on all atoms were below 0.01 eV Å^−1^. The ASE can optionally preserve the original space-group symmetry through constraints. We chose not to use this functionality in order to make the calculations more sensitive to potentially incorrect space-group determination. Test calculations showed that correctly solved structures located near local energy minima do not change space-group symmetry even when optimized in the *P*1 space group.

After the ASE calculations, the CIFs generated in the *P*1 space group were converted back to CIFs with the original space group. The atom labels were also restored, allowing direct comparison based on the correspondence between the original and optimized structures.

### 2.4. Generating descriptors and data for future analysis

The *checkCIF-DFT* software calculates multiple descriptors based on a comparison of the optimized and original CIF structures. The main descriptor is the RMSCD, as defined by van de Streek & Neumann (2010 ▸). Unlike the standard root-mean-square deviation, which is based on atomic displacements, this descriptor employs Cartesian displacements [equation (1)]:
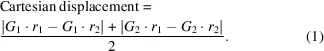
The *r_i_* are the fractional coordinates of the atoms in crystal structure *i*, and *G_i_* is the transformation matrix from fractional to Cartesian coordinates for crystal structure *i*. The use of RMSCD is limited to comparisons of structures with identical definition of unit cells, a condition that is generally satisfied during geometry optimization toward a local minimum. According to its original definition, RMSCD possesses several advantageous properties: it is symmetric with respect to the two structures being compared, varies smoothly under continuous distortions of either one or both structures, and does not require a user-defined parameter such as the number of molecules included in the comparison. These features help mitigate known deficiencies of DFT methods, which are inherited by MLIPs, in reproducing experimental unit-cell parameters. Nevertheless, RMSCD should be evaluated together with changes in the unit-cell parameters, as it is inherently insensitive to unit-cell distortions.

The second most important descriptor is the detection of bond-pattern change, which clearly indicates a fundamental problem. Bonds were identified using a simple distance-based criterion [equation (2)]:
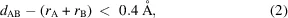
where *d*_AB_ is the interatomic distance and *r*_A_ and *r*_B_ are the covalent radii of atoms A and B, respectively. The tolerance of 0.4 Å was adopted from Artemova *et al.* (2016 ▸). As discussed by Artemova *et al.* (2016 ▸), recommended tolerance values range from 0.4 to 0.45 Å depending on the application and reference source. The value of 0.4 Å has previously been used successfully in the bond-detection engine of the UFF force field. However, this simple bond-detection scheme is not universally reliable, and its limitations are discussed in Section 3.4.

The remaining supporting descriptors are relative changes in cell volume, maximum atomic Cartesian displacement, maximum bond-length change, maximum bond-angle change and maximum torsion-angle change. To enable comparison, cell volumes calculated by MLIP at 0 K were temperature-corrected using an isotropic expansion coefficient of 1.6688 × 10^−4^ K^−1^ (van der Lee & Dumitrescu, 2021 ▸) to correspond to the experimental measurement temperatures. Where applicable, the values were calculated both with and without hydrogen atoms. The main reason for optionally excluding hydrogen atoms is the known difficulty of experimentally localizing them because of their low atomic scattering factor and the common use of older independent atom model refinement rather than Hirshfeld atom refinement (HAR) (Kleemiss *et al.*, 2021 ▸).

**Figure 1:**
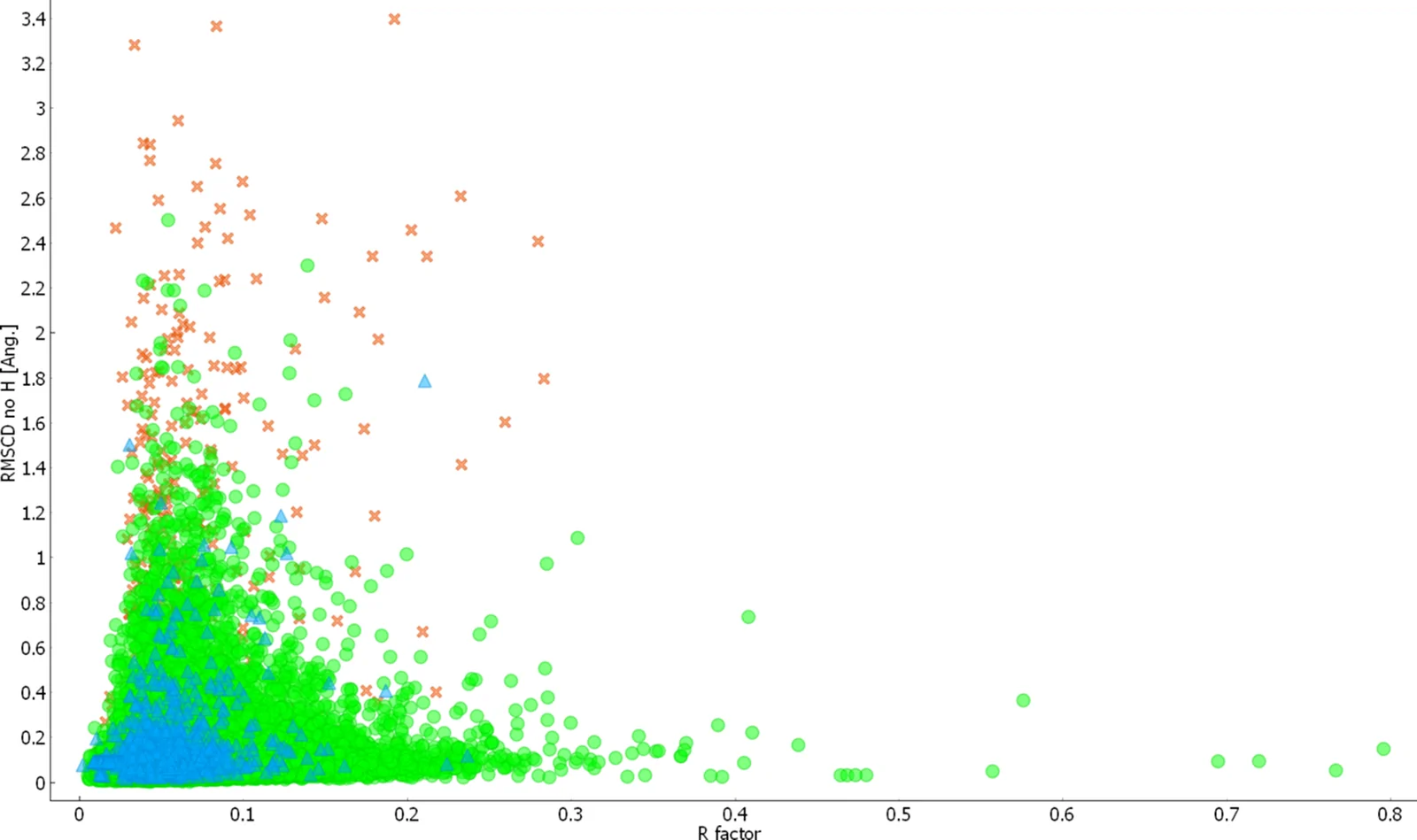
Dependence of RMSCD (excluding H atoms) on *R* factors. Structures with bond-pattern changes not involving hydrogen are marked as red crosses, those with hydrogen-only bond-pattern changes as blue triangles, and those without bond-pattern changes as green circles. Data are shown for 216 917 evaluated structures.

**Figure 2:**
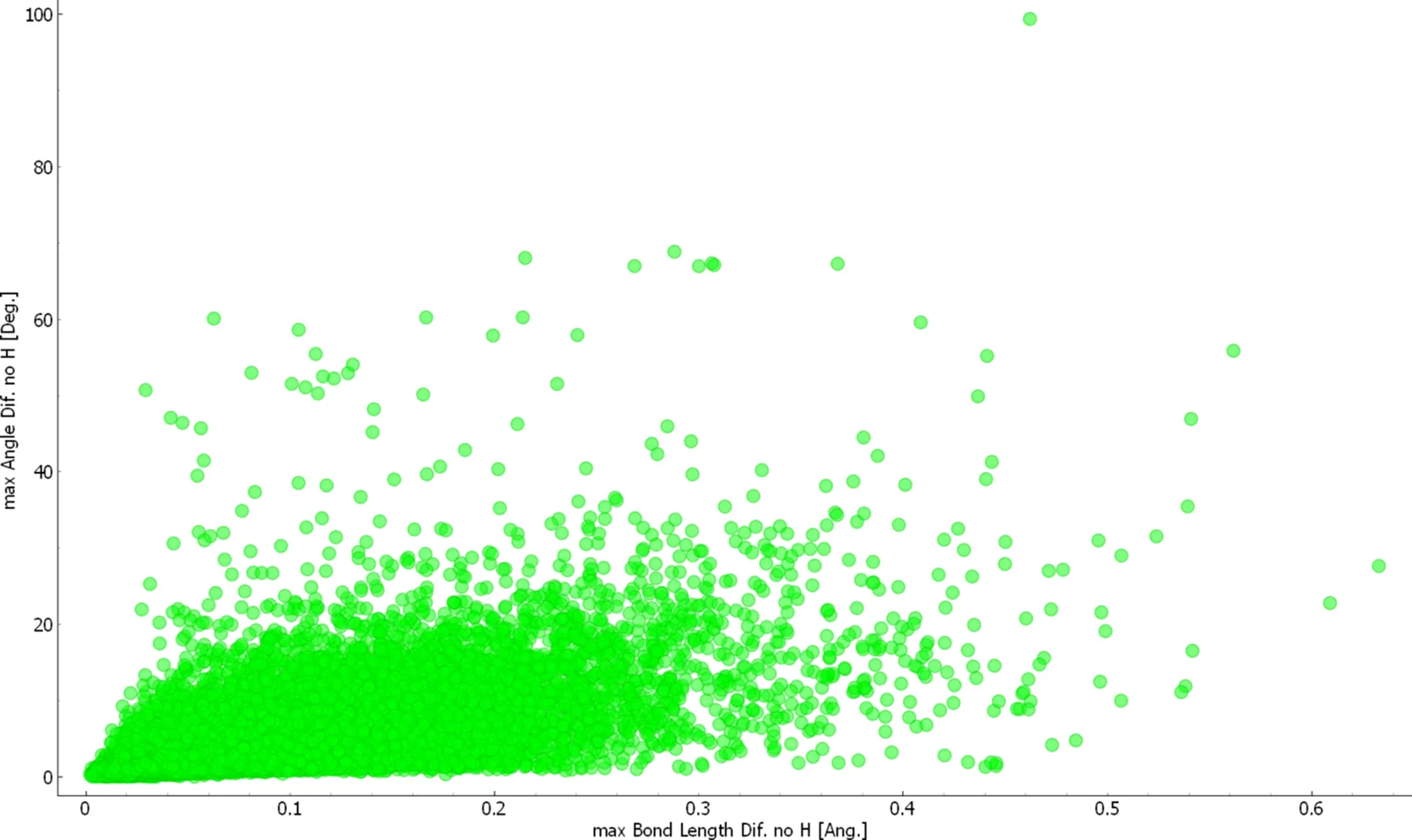
Dependence of the maximum bond-angle difference on the maximum bond-length difference, excluding hydrogen atoms. Data are shown for 215 050 structures without any bond-pattern change detected.

**Figure 3:**
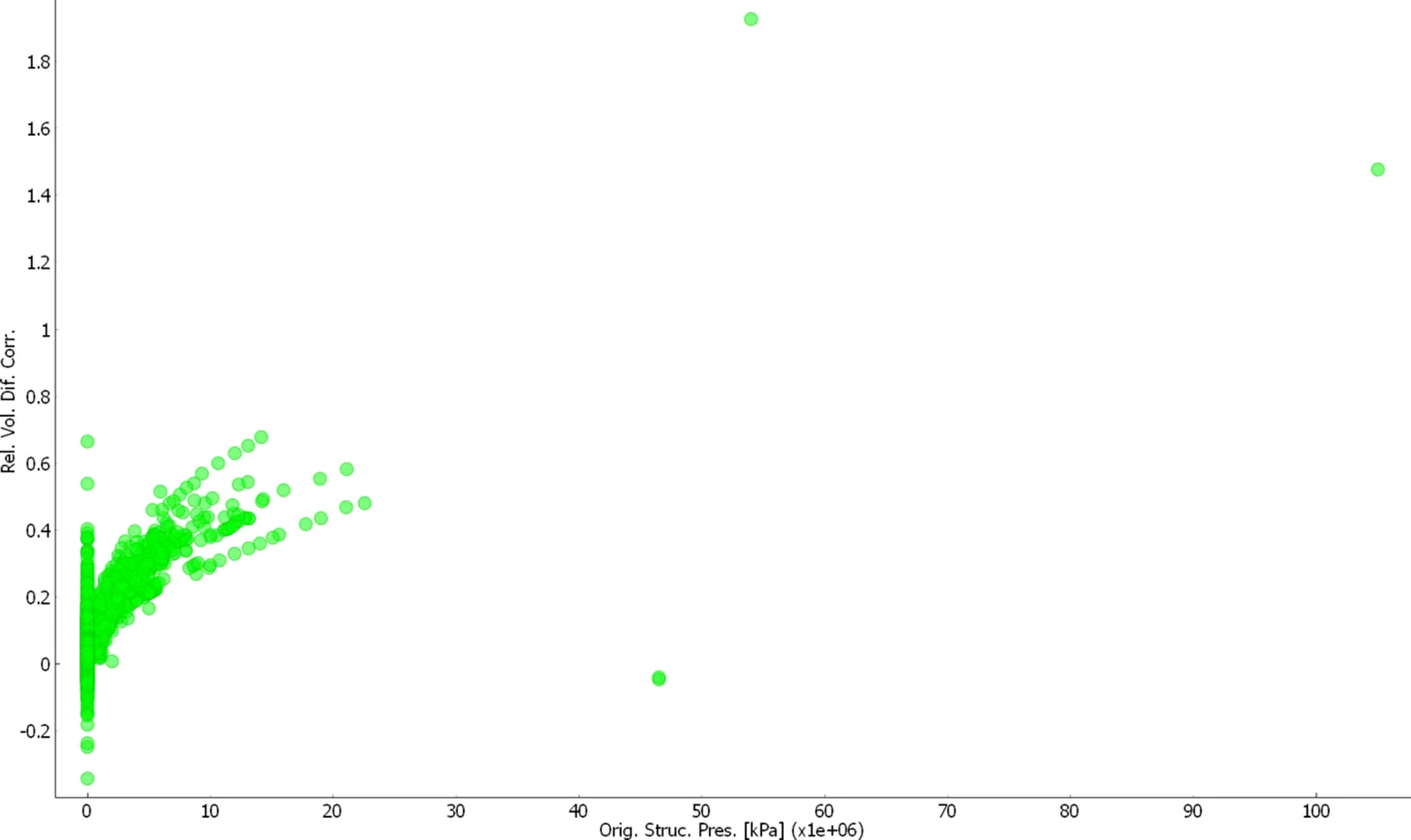
Dependence of the relative difference between the calculated temperature-corrected volume and the experimental volume [(*V*_calc_ − *V*_exp_)/*V*_exp_] on measurement pressure. Data are shown for 215 050 structures without any bond-pattern change detected.

The descriptors were exported in .csv format with markers optimized for direct use in *Orange* software (Demšar *et al.*, 2013 ▸). *Orange* is a general statistical software package with extensive support for data-analysis methods and graphical visualization features. For detecting critical issues, we found that the most illustrative plot was the dependence of RMSCD, excluding hydrogen atoms, on the *R* factor, with bond-breaking detection highlighted in colour and mark shape (Fig. 1 ▸). Structures with RMSCD values greater than 0.25 Å can be expected to be problematic (van de Streek & Neumann, 2014 ▸). Other graphs suitable for problem detection are those showing the maximum bond-angle difference as a function of the maximum bond-length difference (Fig. 2 ▸) and the relative volume difference as a function of the applied experimental pressure (Fig. 3 ▸).

A .csv file containing the CSD codes of all MLIP-processed structures (216 917 in total), together with all the mentioned descriptors, is included in the supporting information. In addition, the file contains an *Orange*-optimized header and additional useful information, such as the space group, *Z*, *R* factor, measurement temperature and pressure.

For further manual inspection, we selected structures with detected bond-pattern change (not involving hydrogen atoms) and structures with RMSCD values greater than 1.0 Å. A sample of 100 structures exhibiting bond-pattern changes involving hydrogen atoms was also inspected manually. Structures with extreme values for other descriptors were also examined manually. Table 3 ▸ summarizes the number of structures flagged by the critical descriptors.

## 3. Results and discussion

Selected issues detected as described in Section 2.4 were manually evaluated. The problem investigation was based primarily on the following information: evaluation of information from the original article, re-refinement of the original data, where available, and structure-geometry optimization using a true high-level DFT method. The most sophisticated method – namely, crystal resynthesis, recrystallization and remeasurement – was not used because of limited human resources. Because the original data, and sometimes even the original article, are often unavailable, DFT optimization is the only option in some cases.

We manually examined the 632 most problematic structures (Table 3 ▸), comprising 467 structures with bond-pattern change not involving hydrogen atoms and 165 structures with RMSCD values greater than 1.0 Å. The detected problems can be divided into four main categories. A very rare problem was detected in the transfer of information from the published CIF to the CIF stored in the CSD database. A larger category of problems is related to true issues with structure determination that had passed all validation methods and the review process. The final group of detected ‘false alert’ is related to the limited ability of the MLIP used to generate a correct PES and to issues with our alert-generating engine. In addition, the extreme but correct behaviour of some molecules can also generate false alerts. The results of the manual analysis for the 632 structures exhibiting bond-pattern changes and high RMSCD are summarized in Table 4 ▸, with a detailed classification provided in Table S3.

For 25 structures with high RMSCD values, we were unable to identify a reliable cause of the discrepancy. Although an element mismatch in both the structure determination and the published data was considered, insufficient evidence was found to support this explanation.

In addition to the detailed manual analysis described above, we manually analysed the first 100 structures from the 1400-structure set showing bond-pattern changes with hydrogen transfer. These results are summarized in Table 5 ▸, with a detailed categorization provided in Table S4.

In the discussion section, we include only the CSD codes of the problematic structures, as providing full references would make the reference list overly extensive. For very commonly detected errors, we give only a few representative examples, as a full list could contain hundreds of structures.

### 3.1. Errors in CIF generation or classification on the CSD side

Issues of this type are typically critical and can even crash processing software because the CIF sometimes contains entirely unexpected content. Examples in this category include structures with no atomic 3D coordinates despite being marked as having 3D coordinates present (LEPCUI, NOYYAE, VENROY, ZISPUO02), and a CIF containing unknown elements (TIGDIW02).

Other issues typically generate bond breaking, including improper transfer of the space group from the original CIF (APOLIB02), S/Si atom mismatch during import from the original authors’ CIF to the CSD (RIRVAP, USOKUJ), and the inclusion of a disordered structure in the set of non-disordered structures (BAQHIM). A notable example is a structure intentionally solved incorrectly as part of Bruker teaching materials (AKEYOF02, YEJLII03, SCCHRN05) but included in the CSD as a correct ‘no errors’ flagged structure.

Sometimes, structures solved from powder data are incorrectly labelled as single-crystal structures (VUDWAU). However, such issues are very rare; therefore, we present examples detected in the entire set of 216 919 prefiltered structures, as well as those identified during prefiltering. We have already partially reported these technical issues to the CSD and will provide an updated list of these clear errors.

### 3.2. Errors in experimental data processing or incomplete CIFs reported by the original authors

#### 3.2.1. Element-type assignment

Incorrect element-type assignment is one of the most critical issues, as it typically leads to bond-pattern change during MLIP processing. A common type of error involves confusion between C, N and O atoms. An example of a structure with a complete mismatch among these elements is IKOVUB, as confirmed by disagreement between the CSD-deposited file and the structure shown in the associated publication.

We also identified cases in which silicon atoms were misassigned as sulfur atoms in tetrahedral SiC_4_ and SiOC_3_ motifs containing only single bonds. For the SiOC_3_ motif in BAKJEG and BUKKID, the element mismatch was confirmed by comparison with the synthesis described in the original articles. For the SiC_4_ motif in AMERAN, the mismatch was confirmed by DFT calculations, as neither the original article nor the experimental data were available.

A search of the entire CSD for SC_4_ and SOC_3_ tetrahedral motifs identified 23 additional structures for which the Si → S mismatch can be confirmed by comparison with synthesis data from the corresponding publications or is highly probable (Table S5).

#### 3.2.2. Missing hydrogen atoms

For correct structure verification using the present methodology, all atoms must be present for all force-calculation methods used; otherwise, the underlying engine cannot correctly identify atom types, hybridization states and electronic structure. Therefore, any missing hydrogen atoms result in bond-pattern change or incorrect molecular conformation and packing, typically detected as high RMSCD values. The absence of hydrogen atoms accounts for the largest fraction (52%) of the detected issues.

The absence of hydrogen atoms is unfortunately common in the CSD, even among structures marked with the ‘no errors’ flag. Although some of these structures were excluded during prefiltering (Section 2.1), fully automatic detection remains technically challenging.

Missing hydrogen atoms range from cases in which all hydrogen atoms are absent (ABOGUW) to more specific omissions, such as missing OH hydrogens (AGUPEZ), NH hydrogens (CEYDER), SH hydrogens (KUDFEV), and, very frequently, COOH hydrogens (ADAGOE). Some cases involve a combination of errors; for example, the incorrect refinement of a disordered *sp*^3^ carbon ring as planar (BUFVEF) followed by incorrect automatic placement of hydrogen atoms.

In nearly all detected cases, the positions of missing hydrogen atoms can be inferred from molecular geometry, hydrogen-bonding patterns, or careful inspection of the electron-density difference map. This suggests that the issue arises from incomplete structural reporting rather than from a lack of available information.

#### 3.2.3. Incorrect hydrogen position

Incorrect hydrogen positions are among the most common causes of high RMSCD values. Geometry-optimization algorithms can in this case locate a local minimum on the PES but not necessarily the global minimum and, therefore, may not rearrange hydrogen atoms correctly.

A typical example is AFINUC, which contains unrealistic hydrogen positions on OH groups. The experimental structure yields an RMSCD of 1.66 Å when compared with the MLIP-optimized structure. After adjusting three OH hydrogen atoms to the expected hydrogen-bonding directions, the RMSCD decreases to 0.09 Å.

In some structures, hydrogen atoms were placed on the basis of incorrect atom hybridization. One example is IFUYEP, which contains a planar geometry for an apparently *sp*^3^-hybridized carbon. In reality, the carbon is *sp*^2^-hybridized and the structure contains an enol motif. In the structure of MAQWEJ, the authors probably forced the hydrogen position to correspond to the formula reported in their article rather than accepting different bond orders and hydrogen locations. In LISLUW, the authors added a non-existent hydrogen atom, generating a four-valent N atom and ignoring the uncompensated charge of the molecule.

#### 3.2.4. High-pressure studies without pressure information

All extreme volume changes, defined as relative volume changes greater than 20%, are related to high-pressure studies (Fig. 3 ▸). Outliers at ambient pressure were related to errors in the pressure description in the CIF. Automatically eliminating such false alerts would require the proper use of the _diffrn_ambient_pressure keyword in the CIF by the authors. The authors of high-pressure studies sometimes omit any mention of high pressure in the CIF (AXUDED01) or do not use the standard kPa units (BNPYRE21).

The very high pressure structures looking like outliers represent a genuine structure of solid nitro­gen (NOBLID03) or a laser-heated high-pressure study of tetra­fluoro­methane (TFMETH06).

### 3.3. Errors related to incorrect MLIP PES generation

Geometry optimization by MLIP cannot give better results than the DFT method used to generate the training model. Unfortunately, the PBE-D3 functional and dispersion correction used for the OMC25 model are known to generate incorrect hydrogen proton transfer (Hušák *et al.*, 2022 ▸). The PBE functional suffers from self-interaction error, which over-stabilizes charged species, and this error is inherited by the MLIP. A lot of structures with bond breaking involving hydrogen atoms fall into this category.

We investigated this issue in detail for the structure with CSD code BAQSUK. After geometry optimization using the selected MLIP, the structure contained unexpected H_3_O^+^ cations. We optimized this model using PBE-D3 in *CASTEP*, and the result confirmed that the functional generates a local minimum on the PES corresponding to the presence of the H_3_O^+^ motif. To verify this result, we re-refined the original structure using HAR. The hydrogen atom was clearly located on the OH group and did not form an H_3_O^+^ motif. After geometry optimization of the H_3_O^+^ model using the higher-level meta-GGA r^2^SCAN + MBD functional (Furness *et al.*, 2020 ▸), the hydrogen atom moved back from H_3_O^+^ to the OH group, in agreement with the experimental data.

This general error in the PES makes the MLIP parameterization used here problematic for salt–cocrystal differentiation and for reliable protonation-state investigation. The consequences are discussed in Sections 4 and 5.

On the other hand, we expected that the predicted COOH-to-N hydrogen transfers were artefacts of self-interaction error as well. To test this hypothesis, we re-refined two structures with available full low-temperature data (BEVZOV and BIEZEQ). In both cases, the difference Fourier maps clearly indicated proton transfer; these structures are not cocrystals but salts and therefore fall into the incorrect hydrogen position category.

Another issue with the PES is related to structures that have low occurrence in the MLIP training set. These include structures with N–Br bonds (EZICEX), S–Br bonds (JOXGEK), I–S bonds (DAXXOQ) and Br–P bonds (ERIWOT). This issue can be generalized to all structures containing halogen–halogen bonds and halogen–phospho­rus or halogen–sulfur bonds. Table S2 provides an overview of the frequency of these types of problematic bonds in the OE62 dataset used for OMC25 MLIP training set generation. The data clearly show that bonds involving Br and I are under-represented.

### 3.4. Errors related to incorrect descriptor calculations

Some extreme behaviour in the molecules studied can generate false alerts, particularly bond-breaking alerts. The simple equation (2) can lead to the improper identification of long bonds. Proper handling would require modifying the elemental covalent radii based on hybridization, which is not yet implemented in our code. Bond tolerance should also depend on the types of atoms involved in the bond. This problem overlaps with the poor optimization behaviour of the MLIP used, as mentioned in Section 3.3. We plan to address these issues in a future code release.

### 3.5. Correct extreme structure geometry

For extremely distorted molecular geometries, incorrect bond detection can occur even for C–C bonds, as in AYIWOX, where a short contact is misinterpreted as a bond, leading to a non-existent bond and an apparent five-valent carbon.

Another example of a correct but extreme structure that generates false alerts is NITPIT. In this structure, a *de facto* four-valent nitro­gen atom is present, but one bond is only a short ionic contact.

### 3.6. Calculation performance analysis

The calculations were carried out in parallel on four independent computers with different GPUs (Table S6 summarizes the hardware specifications and performance). The majority of structures (124 008) were processed on a NVIDIA RTX PRO 4000 Blackwell GPU, achieving an average processing time of 35.5 s per structure. When normalized to the performance of this GPU, the total computational workload corresponds to 89 days of wall-clock time for the complete dataset.

## 4. Possible future improvements to the methodology

### 4.1. Handling disorder

The validation of disordered structures is challenging because they represent a significant proportion of the experimental structures (Table 1 ▸). Using the present methodology, this task could be addressed by validating each possible disorder conformation. Such an approach would require clear identification in the CIF of the disorder group to which each atom belongs. The CIF standard already contains such information; however, the question is whether CIFs exported from the CSD follow this standard and whether the authors correctly generate CIFs with such markers.

### 4.2. The use of other functionals for the model training

In Section 3.3, we presented the issues associated with using inadequate functionals for training-model generation. Future work will require MLIPs trained on periodic systems and on models based on functionals and dispersion corrections that are better than PBE-D3. The fastest approach would be to recalculate the OMC25 dataset using, for example, the r^2^SCAN + MBD functional and to retrain the UMA MLIP on these data. Unfortunately, such work goes beyond the computational resources of our group.

### 4.3. Handling long-range electrostatic interactions

A MLIP optimized for the correct handling of long-range interactions in periodic systems will be required in the future. The fact that MACELES(S) outperformed MACE OFF23(S), MACE OFF23(M) and MACE OFF24(M) (Table 2 ▸) indicates that this is a promising direction. The MLIP should be trained for this purpose on periodic charged systems. The modification should potentially include both electrostatic and dispersion forces.

### 4.4. Extension to more elements

The MACE OFF engine is restricted to ten elements (H, C, N, O, F, P, S, Cl, Br and I). The UMA model trained on OMC25 is restricted to 16 elements (H, Li, B, C, N, O, F, Si, P, S, Cl, As, Se, Br, Te and I). Extending coverage to the full contents of the CSD, and ultimately to the ICSD and COD, will require the inclusion of additional elements.

A promising MLIP candidate covering more elements is the recently introduced MACE-POLAR-1 (Batatia *et al.*, 2026 ▸). This model was trained on the OMol25 dataset (Levine *et al.*, 2026 ▸), which comprises organic compounds containing 83 different elements. The use of hybrid density functionals in the generation of the OMol25 dataset addresses the issue of spurious proton transfer discussed in Section 3.3. Furthermore, the explicit treatment of long-range interactions and electrostatic induction in MACE-POLAR-1 should enable the application of the methodology to crystal structures containing ionized atoms.

### 4.5. Better utilization of space-group information

The used ASE software can work only with periodic structures described in the *P*1 space group. Consequently, the computational complexity of the problem increases with the number of atoms in the whole unit cell, rather than with the number of atoms in the asymmetric unit. Handling this issue would require complex rewriting of the ASE.

As discussed in Section 2.3, the ASE can be used to restrain the space group to the original one. In our approach, optimization in the *P*1 space group may result in symmetry breaking because numerical noise causes small deviations from the exact symmetry of the calculated forces. We observed this behaviour for structures far from local energy minima. In one extreme case of a high-symmetry structure with only three atoms in the asymmetric unit (QAMCAM05, space group *I*43*m*), the origin drift generated a false RMSCD-based alert. In a future version of the code, it may be useful to perform geometry optimization both in *P*1 and in the forced original space-group symmetry. In this case we can use space-group change as another marker of potentially problematic structures.

## 5. Conclusions

The results of our MLIP tests on a subset of the CSD demonstrate the viability of the proposed methodology for the studied category of molecular crystals. The MLIP was able to identify problems with the experimental data interpretation. The method should not be regarded as fully reliable owing to the discussed false positives; rather, it should be viewed as an indicator that a structure may contain an error.

Conversely, the experimental data also helped to detect problems with the MLIP. This raises an important methodological question: is it advisable to benchmark methods only on synthetic quantum-mechanical data and reject experimental data, as discussed by Mata & Suhm (2017 ▸)? Our tests show that this is not the case, and that both quantum-mechanical methods and derived MLIPs should be evaluated for their ability to reproduce experimental data, here specifically experimental atomic positions. When the method used to generate the training data for the MLIP is incorrect, the resulting MLIP will also be unreliable, making the entire process a waste of computational resources. This partially occurred with the use of the PBE-D3-functional-based training model in the tested case.

Another question is whether CSD data can be used as training data for MLIPs. In our test, we demonstrated that this is impossible without non-trivial prefiltering of incomplete and erroneous crystal structures. For MLIP generation, a fully curated and validated subset of CSD data needs to be created. Such a subset should contain all atoms, no disorder, no missing solvent, and, especially, no incorrect atom-type assignments. Special care should be given to hydrogen positions, as these are often responsible for the lattice energy and hold the whole crystal together. The creation of such a subset may require an iterative approach: first, a medium-quality MLIP, such as the one used in this work, could be applied; later, a MLIP trained on the structures prefiltered in the first step could be used.

The processing rate discussed in Section 3.6 demonstrates the suitability of the approach for large-scale database screening and, ultimately, for online web-based structure-validation applications.

## Supplementary Material

Supplementary Tables S1-S6. DOI: 10.1107/S2052252526006949/yc5056sup1.pdf

MLIP comparison descriptors for 216917 structures in. csv format (zipped). DOI: 10.1107/S2052252526006949/yc5056sup2.zip

## Figures and Tables

**Table 1:** Results of structure prefiltering prior to MLIP-based structure verification

Rejection reason	Structures rejected based on given test	Structures left after test
Total structures in CSD 6.00 dataset	0	1371757
Presence of elements other than those supported (H, C, N, O, S, P, F, Cl, Br, I)	853820	517937
Disordered structure	90765	427172
Criteria not satisfied (no errors, not polymeric, 3D coordinates present, single-crystal data)	32241	394931
Not published after 2004	95698	299233
Ionized molecules or charged atoms present	41768	257465
Volume > 4000 Å^3^	17725	239740
3D coordinates not present (not covered by previous check)	4	239736
Solvent-accessible volume > 40 Å^3^	16435	223301
Incorrect space group or supercell detected	1148	222153
Chemically unreasonable valence detected	3981	218172
Atoms labelled with ‘?’ indicating disorder	1253	216919

**Table 2:** The comparison of 20 semi-random selected experimental structures (cleaned by r^2^SCAN/MBD DFT) with the optimization results of different MLIP engines with different parameterizations The values are in RMSCD (Å). Hydrogen atoms are excluded for RMSCD calculation.

	Engine/model							
	MACE	MACE	MACE	MACE	MACE	MACELES	UMA	UMA
Structure CSD code	OFF23(S)	OFF23(M)	OFF23(L)	OFF24(M)	Omol	MACELES(S)	UMA(S) (OMC25)	UMA(M) (OMC25)
ACANIL09	0.0761	0.1010	0.1171	0.1274	0.0533	0.0652	0.0446	0.1180
ADIFUS	0.0843	0.1215	0.0463	0.0370	0.1020	0.0718	0.0446	0.0493
AFIGIG	0.0815	0.1041	0.1712	0.0891	0.0883	0.1184	0.0293	0.0214
AGOMOA	0.3063	0.1169	0.0826	0.1285	0.0883	0.1229	0.1547	0.1673
AHUKAS	0.2025	0.1714	0.1217	0.0945	0.0746	0.1282	0.0868	0.0811
AJESAK	0.1501	0.2250	0.0922	0.1251	0.1434	0.1594	0.0615	0.0790
AJOYUV	0.1202	0.1993	0.1036	0.1036	0.1153	0.1145	0.0816	0.0792
ALOVUV	0.2201	0.1363	0.0952	0.1846	0.0984	0.0962	0.0595	0.0505
ANOGUI	0.2386	0.2308	0.1016	0.1565	0.0884	0.1179	0.0520	0.0456
APAMUB	0.1780	0.3412	0.1528	0.1730	0.1449	0.1871	0.0589	0.0606
AQAZOK	0.1970	0.1380	0.0896	0.2370	0.0674	0.0912	0.0422	0.0518
AQUMEH	0.4553	0.2410	0.1955	0.3833	0.1152	0.1709	0.0850	0.0812
ARIWEF	0.1747	0.1603	0.2615	0.0774	0.1159	0.1159	0.1075	0.1288
ATEXOO01	0.3226	0.1227	0.1045	0.2707	0.0995	0.1584	0.0823	0.1549
ATUTEQ	0.1213	0.0694	0.0797	0.0899	0.0641	0.0652	0.0317	0.0374
AVOBIZ	0.2365	0.1644	0.0688	0.0921	0.0666	0.1435	0.0383	0.0287
AWEMOH	0.0907	0.0860	0.1267	0.0981	0.1872	0.1437	0.0612	0.1421
AWUNOV01	0.1839	0.0863	0.1080	0.1104	0.0829	0.1224	0.0507	0.0534
AXUPUH	0.2516	0.1365	0.0834	0.1038	0.0715	0.2056	0.1109	0.1196
AYONUY	0.1230	0.0869	0.0610	0.0823	0.0639	0.0706	0.0781	0.0592
Average	0.1907	0.1520	0.1131	0.1382	0.0966	0.1235	0.0681	0.0804

**Table 3:** Structures identified as problematic based on descriptor analysis

Problem detection reason	Structures rejected based on given test	Structures left after test
Initial dataset	0	216919
Nonexistent (unknown) element present	1	216918
CIF space group does not correspond to original CIF	1	216917
Bond breaking (not including H atoms)	467	216450
Bond breaking (involving H atoms)	1400	215050
RMSCD > 1.0 Å	165	214885
RMSCD > 0.25 Å	3166	211719

**Table 4:** Categorization of problems identified in the 632 structures exhibiting critical issues

Problem detected	Problem type	Number of structures	% of total structures in category
Missing H atoms	True issue	331	52.37
Bond break or creation with Cl, Br, I involved	False alert	104	16.46
Incorrectly placed H atoms	True issue	46	7.28
Non-realistic bond lengths of non-H atoms	True issue	42	6.65
Unknown issue detected by high RMSCD	Unknown	25	3.96
Other incorrectly detected bond-pattern change	False alert	22	3.48
CSD data classification or labels issue	CSD issue	17	2.69
Element-type mismatch	True issue	15	2.37
High-pressure study positions deformation	False alert	11	1.74
Planar C *sp*^3^ geometry	True issue	8	1.27
Correct extreme C–C bond geometry	False alert	5	0.79
High *R* factor and isotropic refinement	True issue	3	0.47
Unrealistic bond angles of non-H atoms	True issue	2	0.32
Energy-refinement origin drift	False alert	1	0.16

**Table 5:** Categorization of problems identified in the 100 structures exhibiting bond-pattern change with hydrogen involved

Problem detected	Problem type	Number of structures
Unrealistic H transfer	False alert	40
Delocalized H	Alternative interpretation	24
COOH to N transfer (false cocrystals)	True issue	15
Missing H atoms	True issue	13
Unrealistic H-position geometry	True issue	7
CSD data classification or labels issue	CSD issue	1

## Data Availability

Additional data and a file containing structure-validation descriptors are available in the supporting information.
