# Supplementary material for: The use of machine learning interatomic potentials for the verification of experimental molecular crystal structures
Source: IUCrJ. 2026 Jul 27;13(Pt 5):623–34. doi: 10.1107/S2052252526006949 (PMC13548835; doi:10.1107/S2052252526006949)
Supplement: Supplementary file 1 [file m-13-00623-sup1.pdf]

IUCrJ

Volume 13 (2026)

Supporting information for article:

The use of machine learning interatomic potentials for the verification of experimental molecular crystal structures

M. Hušák, F. Fňukal and J. Čejka

Table S1 The comparison of 20 semi-random selected experimental structures with the optimization results of different methods. The values are in RMSCD (Å). Hydrogen atoms excluded from comparison.

Method	Molecular mechanic	DFTB	MLIP	DFT
Method details / Structure CSD code	COMPASS III[a)]	DFTB+[b)]	UMA(S) (OMC25)[c)]	r2SCAN+MBD[d)]
ACANIL09	0.3197	0.1747	0.0316	0.0580
ADIFUS	0.4645	0.5457	0.0667	0.0409
AFIGIG	0.1675	0.1860	0.0988	0.0983
AGOMOA	0.3146	0.1826	0.1095	0.1253
AHUKAS	0.3514	0.2584	0.0329	0.0889
AJESAK	0.1988	0.1796	0.0547	0.0521
AJOYUV	0.5217	0.4119	0.0563	0.1670
ALOVUV	0.2166	0.3349	0.1028	0.1232
ANOGUI	0.2417	0.2236	0.0301	0.0538
APAMUB	0.2603	0.2725	0.1275	0.0667
AQAZOK	0.2478	0.2180	0.0343	0.0786
AQUMEH	0.9038	0.9354	0.0687	0.1829
ARIWEF	0.2505	0.7968	0.0310	0.0689
ATEXOO01	0.3952	0.3854	0.1680	0.0458
ATUTEQ	0.1077	0.1938	0.0360	0.0558
AVOBIZ	0.1688	0.1944	0.0306	0.0523
AWEMOH	0.1722	0.1622	0.0409	0.0570
AWUNOV01	0.1308	0.1482	0.0516	0.0651
AXUPUH	0.2005	0.2729	0.0562	0.1124

AYONUY	0.1846	0.1990	0.0406	0.0488
**Average**	**0.2909**	**0.3138**	**0.0634**	**0.0821**

a) COMPASSIII as implemented in BIOVIA Materials Studio 2022, cell optimized, Ultra-fine parameters set, force filed assigned charges

b) DFTB+ as implemented in BIOVIA Materials Studio 2022, cell optimized, Fine parameters set, D4 dispersion correction, 3ob Slater-Koster library, self consistent charges

c) UMA(S) MLIP in ASE environment, cell optimized, OMC25 model, 0.01 eV/Å maximum force convergence limit

d) r2SCAN functional in CASTEP 23.1 environments, cell optimized, MBD dispersion correction, automatic precise cut-off selection based on on the fly pseudo-potentials, 0.00001 eV/atom total energy tolerance, 0.03 eV/Å maximum force, automatic finite basis correction used

Table S2 Frequency of selected motif occurrences in the OE62 dataset based on CSD refcode analysis.

Element or bond motive present in molecules	Number of molecules with given future	Frequency in dataset in %
N	40837	66.4546
O	49879	81.1687
S	14933	24.3007
P	3606	5.8681
F	4209	6.8494
Cl	7005	11.3993
Br	4337	7.0577
I	832	1.3539
Cl-C	6469	10.5271
Cl-N	28	0.0456
Cl-P	274	0.4459
Cl-Cl	1	0.0016
Br-N	3	0.0049
Br-S	1	0.0016
Br-P	7	0.0114
Br-Br	0	0.0000
I-C	814	1.3246
I-O	2	0.0033
I-P	7	0.0114
I-S	1	0.0016
I-I	0	0.0000

Table S3 Categorization of the structures with bonding pattern change detected with hydrogen excluded (467 structures) and RMSCD higher than 1 Å (165 structures)

Issue reason	Issue characteristic	Number of structures detected based on bonding pattern change	Number of structures detected based on RMSCD higher than 1 Å
Br Br bond creation	false alert	1	0
Br N bond creation	false alert	2	0
Br P bond break	false alert	5	0
C C bond too long	true issue	10	1
C C bond creation from too short contact	true issue	13	0
C C true long bond	false alert	5	0
Cl C bond break	false alert	2	0
Cl C bond creation	false alert	2	0
Cl Cl bond break	false alert	3	0
Cl N bond break	false alert	1	0
Cl P bond break	false alert	7	0
Cl P bond creation	false alert	1	0
Disorder in non disordered structures	CSD issue	11	0
Duplicate C on 2 fold axis	CSD issue	1	0
Element mismatch (C instead of O)	true issue	1	0
Element mismatch (multiple O C N switch)	true issue	1	0
Element mismatch (S instead of Si)	true issue	13	0
I C bond break	false alert	4	0
I I bond break	false alert	5	0
I I bond creation	false alert	8	0
I N bond break	false alert	10	0

I N bond creation	false alert	7	0
I O bond break	false alert	27	0
I P bond break	false alert	3	0
I S bond creation	false alert	16	0
Incorrect C label on symmetry operation	CSD issue	1	0
Intentionally wrong structures	CSD issue	2	0
Missing H (all)	true issue	86	10
Missing H on C	true issue	49	26
Missing H on COOH	true issue	20	2
Missing H on N	true issue	22	22
Missing H on OH	true issue	83	10
Missing H on S	true issue	1	0
N C distance too short	true issue	1	0
Non-existing H on N	true issue	1	1
O C bond too long	true issue	15	0
O O distance too short	true issue	1	0
O S bond creation	false alert	1	0
P C bond break	false alert	3	0
P N bond creation	false alert	3	0
P N extreme correct bond	false alert	3	0
P O bond break	false alert	1	0
P P bond break	false alert	1	0
P P short contact	false alert	6	0
Planer SP3 C geometry	true issue	1	7
S C bond too long	true issue	1	0
S S bond breaking	false alert	3	0
S S bond creation	false alert	1	0

Unrealistic H position on C	true issue	2	0
Unrealistic H position on N	true issue	0	8
Unrealistic bond angles (H excluded)	true issue	0	2
Incorrect H label on symmetry operation	CSD issue	0	1
OH H atoms not in H-bond direction	true issue	0	26
High pressure study with deformed geometry	false alert	0	11
Incorrect H atom to bonded atom assignment	true issue	0	8
Energy refinement origin drift	energy refinement issue	0	1
Powder data solution	CSD issue	0	1
Unknown issue	unknown	0	25
High R factor and isotropic refinement	true issue	0	3

Table S4 Categorization of the 100 sample structures detected by bonding pattern change (hydrogen involved in the change)

Issue reason	Issue characteristic	Number of structures detected based on bonding pattern change
Missing H on C	true issue	3
Missing H on COOH	true issue	1
Missing H on OH	true issue	8
Missing H on Cl	true issue	1
Delocalized H N-H-O	alternative interpretation	5
Delocalized H O-H-O	alternative interpretation	19
Disorder in non disordered structures	false alert	1
H transfer from OH to N	false alert	12
Creating H_3O^+ ions	false alert	21
H transfer from SHE to N	false alert	2
H transfer from N to COOH	false alert	1
Unrealistic H positions	true issue	7
H transfer from COOH to N	true issue	15
Creating -COH_2^+ ionised group	false alert	4

Table S5 Structures in the CSD with silicon atoms incorrectly assigned as sulphur atoms.

SC_4 motive: ALARUA, AMERAN, FOGTOO, GOQTEN, JIBGIO, KIRBEW, NOLXOF, OZACAW

SOC_3 motive: BAKJEG, BUKKID, FEJQIX, HUDTAD, JEBVUK, LOZTUT, QAHHIS, QUFGOP, RIRVAP, SASGUQ, SASHEB, SAZROE, SOMMIU, USOKUJ, WIYNIB

Table S6 Average geometry optimization times for the 20 benchmark structures on different HW (seconds). UMA(S) (OMC25) MLIP used.

Graphic card	NVIDIA RTX PRO 4000 Blackwel	NVIDIA GeForce 3050	NVIDIA RTX A4000	NVIDIA GeForce RTX 5060 Ti
CUDA cores	8960	2304	6144	4608
Memory (GB)	24	6	16	16
ACANIL09	18.07	43.19	22.31	28.87
ADIFUS	22.47	46.33	25.08	35.08
AFIGIG	12.55	30.71	16.84	22.69
AGOMOA	55.63	135.70	67.61	86.12
AHUKAS	38.03	97.22	48.95	60.81
AJESAK	11.90	28.18	16.63	23.78
AJOYUV	37.80	90.59	45.84	57.89
ALOVUV	34.05	84.35	42.05	54.17
ANOGUI	10.86	24.66	15.11	23.50
APAMUB	26.62	65.03	33.47	41.76
AQAZOK	18.73	33.22	20.34	32.31
AQUMEH	42.22	85.75	52.41	71.12
ARIWEF	135.73	328.30	172.45	214.02
ATEXOO01	17.83	43.56	23.18	29.72
ATUTEQ	26.28	63.70	32.48	41.45
AVOBIZ	23.18	48.58	26.92	40.50
AWEMOH	12.85	23.28	16.89	28.25
AWUNOV01	49.49	30.23	59.77	28.26
AXUPUH	66.37	159.26	80.05	105.54
AYONUY	28.91	69.94	34.64	43.76

Average	**34.48**	**76.59**	**42.65**	**53.48**
